# Neuroinflammation in an optimized model of lysophosphatidic acid (LPA)-induced post-hemorrhagic hydrocephalus

**DOI:** 10.21203/rs.3.rs-6762718/v1

**Published:** 2025-06-03

**Authors:** Paloma Sánchez-Pavón, Carter R. Palmer, Christine S. Liu, Valerie P. Tan, Victoria A. Blaho, Jerold Chun

**Affiliations:** Sanford Burnham Prebys Medical Discovery Institute; Sanford Burnham Prebys Medical Discovery Institute; Sanford Burnham Prebys Medical Discovery Institute; Sanford Burnham Prebys Medical Discovery Institute; Sanford Burnham Prebys Medical Discovery Institute; Sanford Burnham Prebys Medical Discovery Institute

**Keywords:** post-hemorrhagic hydrocephalus, lysophosphatidic acid, microglia, single-cell RNA-seq, neuroinflammation

## Abstract

Post-hemorrhagic hydrocephalus (PHH) is a neurological disease that primarily affects premature infants and involves infiltration of blood into the brain’s ventricles followed by excessive accumulation of cerebrospinal fluid (CSF), leading to ventricular enlargement and increased intracranial pressure. The precise mechanisms driving PHH development and persistence are incompletely understood and lack disease-modifying treatments. Using a mouse model of PHH, we have identified transcriptomic, proteomic, and cellular features of PHH involving neuroimmune and neurovascular alterations recapitulating those reported in human disease. Improvement upon a lysophosphatidic acid (LPA)-induced PHH mouse model was combined with unbiased proteomic and single-nucleus transcriptomics that identified microglial molecular pathways propagating PHH. Pharmacological depletion of microglia *in vivo* significantly reduced PHH-associated ventriculomegaly. These data identify microglial and neurovascular elements in the development of PHH, implicating them as other potentially tractable therapeutic targets beyond LPA receptors, towards developing medical treatments for PHH.

## Introduction

Hydrocephalus is a neurological disease characterized by abnormal accumulation of cerebrospinal fluid (CSF) in the brain, leading to expansion of the lateral ventricles and increased intracranial pressure. It is driven by an imbalance between CSF production and clearance [1, 2]. The choroid plexus is a secretory tissue responsible for most CSF production and arises from the pia mater as vascularized invaginations inside brain ventricles [3, 4]. Ependymal cells lining the cerebroventricular walls and the central canal of the spinal cord control CSF diffusion into the central nervous system (CNS) [5]. CSF is typically removed through subarachnoid spaces via arachnoid villi located along the superior sagittal venous sinus, intracranial venous sinuses, and around the roots of spinal nerves [6, 7]. CSF then reaches the venous blood system through the dural venous sinuses via cranial arachnoid granulations and flows into the lymphatic system, passing through the nasal cribriform plate and the perineural sheaths [8, 9]. Additionally, meningeal lymphatic vessels drain CSF into the cervical lymph nodes, which led to the observation of a new mechanism of CSF clearance [10, 11].

Hydrocephalus can be congenital or acquired. Congenital hydrocephalus is caused by brain and spinal cord defects such as spina bifida, aqueductal stenosis, brain malformations, or infections during pregnancy such as rubella. Acquired hydrocephalus is caused by head injuries, brain tumors, intraventricular hemorrhages, and brain infections such as meningitis [12, 13]. Post-hemorrhagic hydrocephalus (PHH) is considered an acquired hydrocephalus and develops in response to an intraventricular hemorrhage (IVH). PHH develops when a grade III or higher IVH occurs in premature infants and blood enters the lateral ventricles, leading to CSF accumulation, lateral ventricle enlargement, and increased intracranial pressure. Up to 35% of all premature infants with IVH will develop PHH [14–23]. Current treatments for hydrocephalus include surgically implanting shunts to drain excess CSF, puncturing the floor of the third ventricle to allow CSF drainage, or cauterizing the choroid plexus to reduce the amount of CSF that is produced [17, 24–29]. These invasive treatments present significant clinical challenges, and no effective medical (as compared to surgical) treatments currently exist.

Mouse models of hydrocephalus have implicated lysophosphatidic acid (LPA: (2R)-2-hydroxy-3-[(9Z)-octadec-9-enoyl] oxypropyl dihydrogen phosphate) in the initiation of fetal hydrocephalus and PHH, recapitulating multiple cellular and neuroanatomical hallmarks of human PHH [30, 31]. LPA is a bioactive lysophospholipid that signals through a family of six G-protein coupled receptors (GPCRs): LPA_1–6_ [32–35]. It is present in multiple body fluids including plasma and CSF, with concentrations varying from nanomolar range under physiologically normal conditions to micromolar range following hemorrhagic events [30, 31, 36–44]. Intrauterine intraventricular injection of LPA in embryonic day 13.5 (E13.5) mice was demonstrated to be sufficient to induce fetal hydrocephalus [30]. Subsequently, it was shown that LPA injection in the lateral ventricles of mice at postnatal day 8 (P8), an age that correlates with viable (intensive care admission) premature birth in human neonates, also phenotypically reproduced PHH that included increased intracranial pressure, CSF accumulation, and ependymal cell damage involving phagocytic cells including microglia and macrophages [31].

Towards identifying other medically tractable therapeutics beyond LPA receptor modulators, which remain unvalidated in human PHH despite animal model efficacy, we focused on the possible tractability of neuroinflammation-related targets as supported by the involvement of innate immune microglia and macrophages [31]. Using an optimized LPA-induced PHH mouse model that recapitulates key characteristics of PHH, including ventriculomegaly and CSF overaccumulation, we examined neuroinflammatory pathways described in the literature as causing choroid plexus CSF hypersecretion [16, 45–47]. Cellular and molecular analyses employed here identified neuroimmune and related neurovascular changes in brains of LPA-injected pups, further linking receptor-mediated LPA signaling to PHH and representing possible therapeutically tractable pathways towards developing new medical treatments of PHH by targeting neuroimmune mechanisms.

## Methods

### LPA handling: Preparation and storage

18:1 Lyso PA (LPA) in chloroform (25mg) was purchased from Avanti Polar Lipids and shaken vigorously prior to opening the glass vial, then rapidly aliquoted into glass vials with glass micro-inserts to reduce adherence to the vial walls. Each vial was aliquoted at the desired concentration (5 mM or 1 mM) and dried out for 5 minutes in a vacuum-sealed centrifuge. Dried LPA was stored at −20°C covered in parafilm. LPA was reconstituted in 0.01% fatty acid-free bovine serum albumin (BSA) in phosphate-buffered saline (PBS) and sonicated using a Qsonica Q125 for 2.5 minutes, 5 seconds on and 3 seconds off at 80% amplitude.

#### Surgery

P7 pups (C57BL/6 S-56 mice) were used in these experiments. Mice were anesthetized for five minutes with gaseous isoflurane. Subsequently, they were placed on a stereotactic frame that was adapted for the pups. After properly disinfecting the area, an incision was made on the top of the head to expose the skull. 5 μl of LPA or vehicle (0.01% BSA) were drawn into a 10 μl NanoFil syringe (World Precision Instruments) and injected into the right brain lateral ventricle by inserting a 35 gauge needle from the bregma, 3.2 mm caudal by 0.7 mm lateral by 1.2 mm deep. After surgery was complete, the incision was sealed with glue and the pups were placed in a closed holding cage on top of a heating pad for recovery. Postoperative flunixin meglumine (Banamine, 1 mg/kg) was administered subcutaneously for 2 days for pain management. When the pups regained consciousness, they were placed back with their mother. Excessive grooming from the mothers occurred frequently; therefore, it was necessary to monitor the pups and close the head wounds again if needed until the day of tissue collection. If the mothers showed signs of extreme aggressiveness, the pups were placed with foster mothers.

#### Histology: Paraffin embedded sections

Whole mouse brains were harvested at P15, 8 days after surgery. Brains were fixed overnight in scintillated glass vials containing 15 ml of formalin acid alcohol (4% formaldehyde and 5% glacial acetic acid in 70% ethanol). They were processed in a Tissue-Tek VIP, and embedded in paraffin with a Tissue-Tek TEC. Coronal sections were cut using a Leica microtome at 10 μm thickness throughout the ~ 200 μm span of the lateral ventricles. The slides were then deparaffinized in 3 subsequent xylene washes, followed by 3 washes in 100% ethanol and 4 consecutive washes in different concentrations of ethanol (95%, 70%, 50%, and 30%). The slides were then stained in hematoxylin and eosin (H&E), followed by additional ethanol washes in reverse order (2 washes in 70%, 2 washes in 95%, and 2 washes in 100%). Finally, they were cover slipped and preserved in Cytoseal 60.

#### Imaging of H&E sections and lateral ventricle volume quantification

Stained slides were imaged using the Leica Aperio ScanScope. Images were taken at 20X. Lateral ventricle volume was analyzed using the Aperio eSlide Manager, drawing around the area of empty space that corresponds to the lateral ventricles (left and right). All ventricular areas analyzed were then summed and multiplied by 200 μm to calculate the volume.

#### immunofluorescence

We performed transcardiac perfusion with ice cold PBS (3 ml) on P15 mouse pups. Brains were subsequently extracted and placed in 10% neutral buffered formalin (NBF) at 4°C for overnight fixation followed by sucrose cryoprotection in 15% sucrose at 4°C and 30% sucrose. They were then embedded in NEG50 and kept at −20°C until use. Brains were cut using a Leica cryostat at 10 μm thickness, sections mounted on Superfrost Plus slides, and stored at −20°C until staining. Slides were washed with Tris-buffered saline (TBS) and TBS with 0.1% Tween 20, 5% normal donkey serum and incubated with primary antibodies at 4°C. After secondary antibody incubation, slides were mounted in Vectashield with DAPI. Antibodies used included Iba1 (Fujifilm/Wako 019–19741) for detection of macrophages/microglia and GFAP (Neuromics CH22102) for detection of astrocytes.

### CSF extraction

Mice were injected with LPA or vehicle. Vehicle-injected mice were sent to the Animal Models Core Facility at Scripps Research for CSF extraction from the cisterna magna at P15. CSF was extracted from hydrocephalic P15 mouse pups via direct aspiration from enlarged ventricles at Sanford Burnham Prebys. Pups were anesthetized with Avertin (250 mg/kg body weight) via intraperitoneal injection. Surgical procedures did not begin until the mouse was adequately anesthetized, as determined by unresponsiveness to toe pinch. The mouse was secured in a stereotaxic frame (Stoelting) and a skin incision was made in the midline over the skull. The skull was exposed and sterilized with 70% ethanol. A sterile single-use insulin syringe with 31G needle (BD 328291) was padded with tape to facilitate attachment to the syringe holder of the stereotaxic frame. The needle was zeroed at Bregma, and the syringe plunger was pulled back slightly to create negative pressure before entering the enlarged ventricles of the hydrocephalic brain at coordinates (1.0, −0.5, 1.8). CSF was slowly collected, and the needle was immediately withdrawn if blood traces were observed in the aspirate. The extract was transferred into a 1.5 ml microfuge tube, contaminants were pelleted by centrifugation, and the clear supernatant (CSF) was transferred to a sterile tube for downstream analyses.

#### Proteomics

Tandem Mass Tag Protein and Peptide Labeling (TMT) was performed on CSF samples (5 vehicle-injected and 5 LPA-injected) at the Proteomics Core at Sanford Burnham Prebys (SBP). To obtain adequate volumes for the vehicle-treated group, CSF from three mice was pooled for each sample. CSF from LPA-injected mice were individually processed. Data analysis was performed by the Proteomics Core.

#### Cytokine/chemokine analysis

CSF samples from mice injected with LPA or vehicle were sent to Eve Technologies for Mouse Cytokine/Chemokine 44-Plex Discovery Assay Array. The cytokines and chemokines tested were Eotaxin, Erythropoietin, 6Ckine, Fractalkine, G-CSF, GM-CSF, IFNB1, IFNγ, IL-1α, IL-1β, IL-2, IL-3, IL-4, IL-5, IL-6, IL-7, IL-9, IL-10, IL-11, IL-12p40, IL-12p70, IL-13, IL-15, IL-16, IL-17, IL-20, IP-10, KC, LIF, LIX, MCP-1, MCP-5, M-CSF, MDC, MIG, MIP-1α, MIP-1β, MIP-2, MIP-3α, MIP-3B, RANTES, TARC, TIMP-1, TNFα, and VEGF-A. LPA samples were individually collected, but multiple vehicle samples had to be pooled together to reach the necessary volume to perform the assay.

### Nuclei isolation and generation of amplified cDNA libraries

Mice injected with LPA or vehicle were perfused with Hanks’ Balanced Salt Solution (HBSS) and RNAse inhibitors (0.2%) (Takara). Whole brains (without olfactory bulbs and the cerebellum) were stored at −80°C. Frozen brains were immediately submerged in 1 ml of nuclei isolation buffer (20 mM Tris, 320 mM sucrose, 5 mM CaCl_2_, 3 mM Mg(Ac)_2_, 0.1 mM EDTA, 0.1% Triton-X 100, 0.2% RNase inhibitors (Takara Bio recombinant RNase Inhibitor). Extracted nuclei were washed twice in PBS + 0.25mM EGTA + 1% BSA + 0.2% RNase inhibitors (Takara Bio, Mountain View, CA), and resuspended in PBS + EGTA (250μM) + 1% BSA + 0.2% RNase inhibitors + 1.25 μg/ml 4’,6-diamidino-2-phenylindole (DAPI) (Sigma, St. Louis, MO) + NeuN antibody (1:500, Abcam) to label neuronal populations. Stained nuclei were sorted on a FACSAria Fusion (BD Biosciences, Franklin Lakes, NJ) gating for NeuN-negative populations, and collecting non-neuronal nuclei. Samples were kept on ice and processed immediately after sorting. Sorted nuclei were diluted to ~ 700–1,500 nuclei/ml, and a final concentration was confirmed using the Countess 2 Automated Cell Counter. The 10x Genomics Single Cell 3’ v3 kit was then used to prepare samples targeting 10,000 profiled cells.

### Short-read snRNA-seq data processing and QC

10x Genomics CellRanger software (v4.0.0) was used to demultiplex samples, align reads, quantify unique molecular identifiers (UMIs), and generate cell count matrices. Default parameters were used, with the exception of a pre-mRNA reference file (mm10), to capture intronic reads originating from pre-mRNA species present in the nuclei. Using Seurat (v4.0.4), sample matrices were filtered, removing nuclei expressing fewer than 300 genes, containing greater than 1% of reads mapping to mitochondrial RNA, and exceeding an outlier cutoff of UMIs determined by calculating the interquartile range of detected UMIs. Datasets were individually normalized using Seurat’s SCTransform() function.

### Integration, cell type identification, and UMAP visualization

Datasets normalized by SCTransform() were integrated in Seurat, using functions PrepSCTIntegration(), FindIntegrationAnchors(), and IntegrateData(). Zeisel et al.’s dataset [48] was used as a reference with Seurat’s TransferData() function to label cell types in our samples by Class (Astrocytes, Ependymal, Immune, Neurons, Oligos, PeripheralGlia, Vascular). Cells with a maximum confidence score less than 0.8 for the Class label were removed. The integrated data was then scaled and UMAP embeddings were generated.

### Differential Gene Expression (DEG) analysis

Seurat was used to identify differentially expressed genes (DEGs) between cell types in LPA- and vehicle-injected samples. Default parameters were used with FindMarkers() to identify DEGs that were expressed in at least 10% of either of the populations being compared, had at least 0.25 log_2_fold difference, and were significant based on a Wilcoxon Rank Sum test.

### Subclustering immune cells

Immune cells were subset from the total dataset and reintegrated. FindAllMarkers() was used to identify marker genes for the resulting subclusters, and clusters that expressed comparable markers were grouped together. Differential gene expression analysis for the identified subtypes of immune cells (Homeostatic Microglia, Activated Microglia, and Macrophages) was performed using FindMarkers() to identify genes that were differentially expressed between LPA- and vehicle-injected samples.

### Data availability

Fastq files and cellranger output matrices have been deposited at GEO, accession number: GSE272062.

### Pexidartinib (PLX3397)-induced microglia depletion

Pexidartinib (PLX3397, Selleckchem) was freshly prepared immediately prior to injection by dissolving in 5% dimethyl sulfoxide (DMSO) + 45% polyethylene glycol (PEG) 300 + 5% Tween 80 + ddH2O. Pups were injected subcutaneously (50 mg/kg) daily from P1 to P15. Their fur progressively whitened, indicative of a successful depletion [49].

## Results

### Establishing key parameters of the PHH model

**Figure 1:**
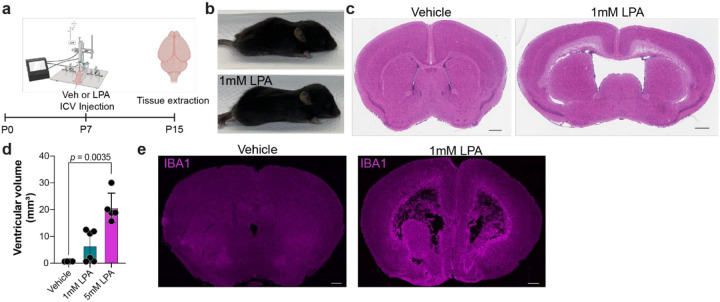
Microglial disruption in a mouse model of LPA-induced post-hemorrhagic hydrocephalus (PHH). (**a**) Schematic of PHH-inducing LPA injections. At 7 post-natal days (P7), pups were injected intracerebroventricularly (ICV) with either vehicle or LPA. 8 days later (P15), pups were euthanized and tissues removed for further analyses. (**b**) Photos of cranial swelling observed at P15 after vehicle (top panel) or 1mM LPA (bottom panel) injection. (**c**) Representative photomicrographs of hematoxylin and eosin (H & E) staining of coronal brain sections from vehicle- or 1mM LPA-injected mice at P15. Scale bars, 600 μm. (**d**) Quantification of ventricular volume eight days post-vehicle, −1mM LPA, or −5 mM LPA injection. Bars represent mean ± s.d and circles represent values from individual mice. *p*-value from ANOVA with Kolmogorov-Smirnov post-test. (**e**) Representative IBA1 staining of vehicle- and 1mM LPA-injected brains at P15. Scale bars, 500 μm.

Previous studies demonstrated that intraventricular injection of LPA at different developmental stages can lead to the development of hydrocephalus [30, 31]. To improve upon previous PHH models, subtle, yet important changes were needed to more effectively simulate human PHH. Specifically, 0.01% bovine serum albumin (BSA) was used as the carrier for LPA, permitting a decrease in injected LPA concentrations from 5mM to 1 mM, resulting in a more physiologically relevant effective concentration. Injections were performed on postnatal day seven (P7) rather than P8 to more closely align with human development in premature infants [50, 51] (Fig. 1a). This modified LPA-induced PHH mouse model reproduced the primary hallmarks of human PHH: ventricular CSF accumulation and the resultant swelling and deformation of the mouse pup head (Fig. 1a), enlargement of the brain lateral ventricles (Fig. 1a), and quantifiable ventriculomegaly (Fig. 1d).

Characterization of previous PHH models detected damage to the ependymal cells lining the lateral ventricles as early as three hours after LPA injection and recruitment of macrophages and microglia to the injured area within six hours of LPA injection [31]. Similar macrophage recruitment and microglial activation was seen in the current model using immunofluorescent detection of IBA1 expression (Fig. 1e) [52]. IBA1 immunolabeling revealed recruitment to the ventricles and a dramatic elevation in IBA1, a sign of microglial activation, as demonstrated by elevated staining brightness eight days post-LPA injection.

### Sustained molecular alterations to CSF composition after LPA injection

**Figure 2:**
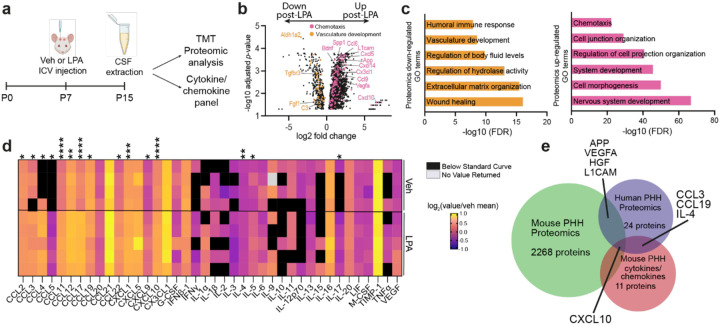
Altered CSF protein concentrations during LPA-induced post-hemorrhagic hydrocephalus (PHH). (**a**) Schematic of CSF collection at P15 after PHH induction by 1mM LPA-injection at P7. (**b**) Volcano plot of differentially regulated proteins in a proteomic assessment of CSF. LPA data are from individual animals, vehicle data are from samples created by pooling CSF from three individual mice. *n* = 5 per group. (**c**) GO terms associated with up- and down-regulated proteins in the CSF. (**d**) Quantification of CSF chemokines and cytokines as measured by the Mouse Cytokine/Chemokine 44-Plex Discovery Assay Array (MD44), expressed as the log2-transformed value normalized to the vehicle mean. Black boxes indicate that the sample measurement was below the assay range, and gray boxes indicate no value was returned. Each row represents data from an individual sample. LPA values are from individual animals, vehicle values are from samples created by pooling CSF from three individual mice. Vehicle-injected, *n* = 4; LPA- injected, *n* = 5. *p < 0.05, **p < 0.01, ***p < 0.001, ****p < 0.0001versus vehicle-injected controls, as determined by unpaired Student’s *t*-test. (**e**) Overlap of proteins identified in human hydrocephalic CSF, list compiled from [58–65] (Online Resource 4), mouse model cytokine and chemokine analysis, and mouse model bulk proteomic assessment. Listed proteins follow the same trend in human and mouse analyses.

To identify potential protein mediators involved in the propagation of PHH inflammation, CSF was extracted from vehicle- and LPA-injected mice and subjected to broad proteomic analysis via Tandem Mass Tag (TMT) isobaric labeling quantitative analysis and a sensitive cytokine/chemokine array (Fig. 2a). Proteomic analysis identified more than 2,000 proteins that were significantly dysregulated in the CSF eight days after LPA injection (P15) (Fig. 2b). Subsequent gene ontology (GO) analysis [53, 54] revealed that 1 mM LPA injection altered the abundance of proteins involved in various pathways. While proteins related to nervous system development and cell-cell junctions were upregulated, those involved in vascular development, wound healing, and body fluid regulation were decreased in abundance. Proteins in the key immune response pathways of chemotaxis and humoral immunity were also significantly affected in the CSF eight days post-LPA injection, implicating dysfunctional immune and vascular responses in LPA-induced PHH (Fig. 2c).

Greater sensitivity for cytokine/chemokine detection was obtained by Discovery Assay Cytokine Array analysis of CSF (Fig. 2d). Of the 44 proteins profiled, 15 were significantly increased in CSF eight days post-LPA injection. Many were proteins considered to be pro-inflammatory chemokines involved in the recruitment of innate immune cells (neutrophils, macrophages, eosinophils, or basophils): CCL2 (MCP-1), CCL3 (MIP-1α), CCL4 (MIP-1β), CCL5 (RANTES), CCL11 (Eotaxin-1), CCL12 (MCP-5), and CXCL1 (KC) [55]. CCL3, CCL4, and CCL5 are also involved in natural killer (NK) cell migration, as well as modulation of T cell interactions with antigen presenting cells [55]. Other significantly increased chemokines are known to regulate T cell migration and subsequent immune responses: CXCL9 (MIG), CXCL10 (IP-10), CCL17 (TARC), CCL19 (MIP-3β), and CCL22 (MDC) [55]. While 13 chemokines were significantly increased, only three of 24 assayed cytokines were significantly affected: IL-4, IL-5, and IL-17. While IL-17 is considered pro-inflammatory, IL-4 and IL-5 are T helper-type 2 (T_H_2) cytokines, involved in responses to tissue damage and pathogens as well as pro-resolution pathways [56, 57].

We then compared the results of our mouse PHH model with those from studies of human hydrocephalus, wherein CSF was analyzed by either proteomics or multiplexed ELISA. Out of 24 biomarkers examined, 22 appeared to be upregulated in the CSF of these patients (VEGFA, HGF, L1CAM, APP, CXCL-10, CCL-3, IL-6, IL10, NCAM1, IL-8, IL-18, MMP-7, MMP-9, IL-1α, IL-1β, IL-12, TGF-β1, IL-4, sFas, SP-G, CCL-19, TNFα) and two appeared to be downregulated (XCL1, TIMP4) [58–65] (Online Resource 4). Five of these proteins (VEGFA, HGF, L1CAM, APP, CXCL-10) were also detected as dysregulated in our proteomics data set from mouse CSF (Fig. 2e). CXCL10 (IP-10) appeared upregulated via human and mouse proteomic analysis as well as via cytokine array and may play an integral role in the recruitment of T cells to sites of PHH-induced inflammation.

### Single-nucleus RNA-sequencing of hydrocephalic brains identified immune and vascular-mediated alterations

**Figure 3:**
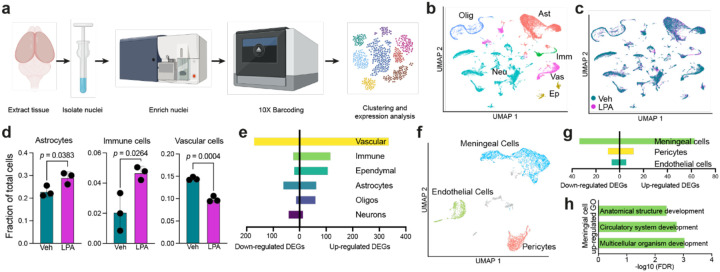
Single-nucleus RNA-sequencing (snRNA-seq) analyses of LPA-induced post-hemorrhagic hydrocephalus (PHH) brains. (**a**) Schematic of snRNA-seq processing of PHH brain nuclei (**b**) Cells were clustered into six cell types: astrocytes (Ast), ependymal cells (Ep), immune cells (Imm), neurons (Neu), oligodendrocytes (Olig), and vascular cells (Vas). Colors indicate cell types. (**c**) UMAP of cell populations identified in (b) colored according to vehicle (blue) or LPA (pink) injection. (**d**) Contribution of astrocytes, immune cells, and vascular cells to the total cell number in vehicle- (green) and LPA-injected (purple) brains. Bars represent mean ± s.d and circles represent values from individual mice. *p*-values from unpaired Student’s *t*-test. (**e**) Differentially expressed gene (DEG) counts in the six cell types observed in (b). (**f**) UMAP of vascular cell-associated subpopulations: pericytes, meningeal cells, and endothelial cells colored according to cell type. (**g**) DEG counts for vascular cell subtypes identified in (f). (**h**) GO terms for upregulated meningeal cell DEGs.

To profile hydrocephalic brain cell types and their activation statuses in an unbiased manner, singlenucleus RNA-sequencing (snRNA-seq) was utilized. After removing the cerebellum and the olfactory bulbs, nuclei were isolated, purified, and processed using the 10x Genomics 3’ Gene Expression kit (Fig. 3a). Nuclei were clustered, and labels were transferred from a reference dataset [48], allowing for the identification of six major cell populations: astrocytes, ependymal cells, immune cells, neurons, oligodendrocytes, and vascular cells (Fig. 3b). UMAP (uniform manifold approximation and projection) plots of nuclei derived from vehicle- versus LPA-injected animals showed similar, mostly overlapping cell type clusters between the two groups (Fig. 3b). Initial analysis revealed changes in the proportions of different cell types present in the brain in response to LPA; whereas astrocytes and immune cells were significantly elevated, vascular cells were significantly reduced after LPA injection (Fig. 3d). Proportions of ependymal cells, neurons, and oligodendrocytes did not change significantly in response to LPA (**Online Resource 1**).

Further analysis revealed changes in gene expression within the six cell types when comparing nuclei from LPA-injected pups to vehicle controls. Differentially expressed genes (DEGs) were defined as those expressed in at least 10% of either LPA-injected or control populations, ≥ 0.25 log_2_ fold-change, and *p* < 0.05 by Wilcoxon rank sum test with Bonferroni correction. In response to LPA injection, vascular cells had the greatest number of up- and down-regulated genes (Fig. 3e, **Online Resource 5**). Vascular dysfunction is believed to be a likely driver of hydrocephalus, as it may prevent effective draining of excess CSF. To investigate further, vascular cells were subclustered into pericytes, meningeal cells, and endothelial cells (Fig. 3f). Subclustering revealed that meningeal cells drove the obvious vascular transcriptomic changes, as they had dramatically more DEGs (97) compared to only 22 in pericytes and 13 in endothelial cells (Fig. 3g). Ontological assessment of these upregulated meningeal cell genes pointed to abnormal developmental processes (Fig. 3h), likely from compensatory reformation of functional structures following significant structural disruption observed in the brain [53, 54].

### Distinct activated microglial profiles identified in hydrocephalic brains after LPA injection

**Figure 4:**
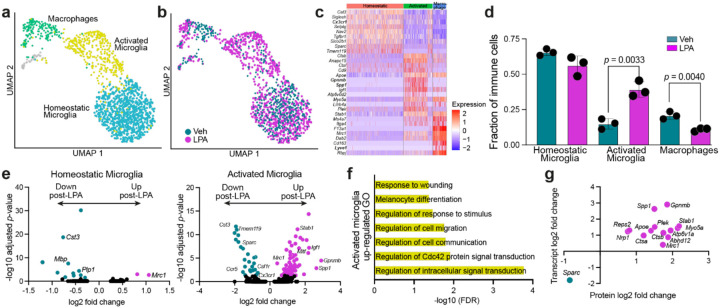
Transcriptomic detection of microglial activation in LPA-induced post-hemorrhagic hydrocephalus (PHH) compared to controls. (**a**) UMAP of immune cell subpopulations from PHH vs. control brains at P15: homeostatic microglia, activated microglia, and macrophages, colored according subtype. (**b**) UMAP of immune cell populations identified in (a) colored according to vehicle- (green) or LPA-injection (purple). (**c**) Heatmap of key immune cell subtype-identifying genes. (**d**) Contribution of homeostatic microglia, activated microglia, and macrophages to the total immune cell number in vehicle- (green) and LPA-injected (purple) brains. Bars represent mean ± s.d, and symbols represent values from individual mice. *p*-values from unpaired Student’s *t*-test. (**e**) Volcano plots showing key DEGs from homeostatic and activated microglia. Genes that meet DEG cutoffs are colored green if downregulated after LPA injection or purple if upregulated after LPA injection. (**f**) Top GO terms associated with genes upregulated after LPA injection in activated microglia clusters. (**g**) Genes differentially regulated in the same direction in both CSF proteomic data and activated microglia transcriptomes.

As our proteomics and cytokine array data indicated a sustained neuroinflammatory response to LPA injection, immune cells were subclustered to identify specific cell types (Fig. 4a). Three populations were defined, with observable separation between vehicle- and LPA-injected brains (Fig. 4b). One population was determined to be peripheral macrophages based on the presence of markers including *Lyve1, F13a1*, and the lack of microglia-specific marker *Cx3cr1* (Fig. 4c). The other two populations, termed homeostatic microglia and activated microglia, both expressed microglial markers, including *Cx3cr1*, but were distinguished by the absence or presence of markers for microglial activation, including *Spp1, Apoe*, and *Gpnmb* (Fig. 4c). While peripheral macrophages were significantly decreased at this time point, activated microglia accounted for significantly more of the total immune cell population, and homeostatic microglia were unchanged in response to LPA injection (Fig. 4d).

The significant increase in activated over homeostatic microglia coincided with a change in gene expression in the activated microglia. While macrophages were determined to have a single DEG (**Online Resource 6**) and homeostatic microglia had 14 (Fig. 4e, **Online Resource 6**), the transcriptome of activated microglia was dramatically altered. This cell subpopulation had 91 DEGs, including further increases in activation markers and loss of homeostatic markers (Fig. 4e, **Online Resource 6**). To further characterize the differences between activated microglia from LPA- and vehicle-injected brains, GO analysis was performed. Pathways related to signal transduction, cell migration, and response to wounding were impacted the most (Fig. 4f). These data correlate with data obtained from our earlier model, which showed that microglia were activated and actively recruited to the ventricular borders in response to LPA-induced hydrocephalus [31]. We next compared the single-nucleus transcriptomic data from activated microglia with our proteomic data from bulk brain tissue. This analysis identified 14 genes that were similarly altered at both the mRNA and protein levels (Fig. 4g), including microglial activation markers, *Spp1* and *Gpnmb*. By contrast, homeostatic microglia had only a single gene with coordinated mRNA-protein dysregulation, and macrophages had none. Collectively, these data suggest that both a significant increase in the number of activated microglia and a heightened state of activation persisting a week after LPA injection contribute to the ongoing inflammatory hydrocephalic status.

### Sustained microglia depletion diminishes ventriculomegaly

**Figure 5:**
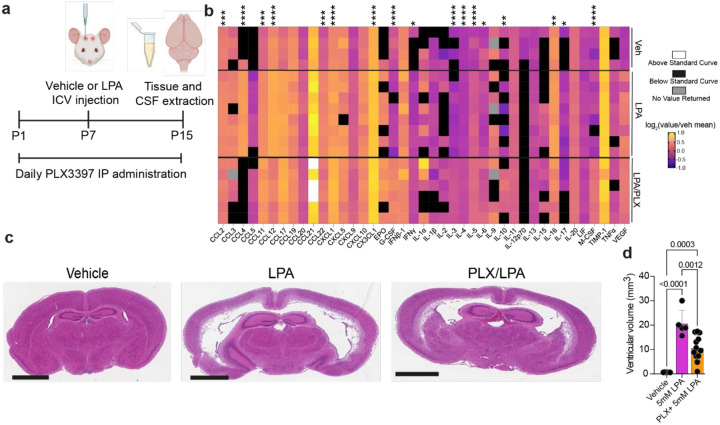
Microglial depletion by PLX3397 ameliorates CSF cytokine abnormalities and ventriculomegaly in LPA-induced post-hemorrhagic hydrocephalus (PHH). (**a**) Schematic of microglia depletion induced by daily subcutaneously PLX3397 administration in the PHH model. (**b**) Quantification of chemokines and cytokines in CSF as measured by the mouse cytokine/chemokine 44-Plex Discovery Assay (MD44) expressed as the log_2_-transformed value normalized to the vehicle mean. Black boxes indicate that the sample measurement was below the assay threshold, white boxes signify the measurement was above the assay threshold, gray boxes indicate that a reading was not obtained. Each row represents data from an individual sample. LPA (n=8) and LPA/PLX3397 (n=6) values are from individual animals; vehicle values are from samples created by pooling CSF from three animals. Asterisks denote significant differences between LPA and LPA/PLX3397 as determined by one-way ANOVA followed by post-test with Tukey’s correction for multiple comparisons. *p < 0.05, **p < 0.01, ***p < 0.001, ****p < 0.0001. (**c**) Representative photomicrographs of hematoxylin and eosin (H & E) staining of coronal brain sections from vehicle-, 5 mM LPA-injected, and PLX3397-treated, LPA-injected mice at P15. Scale bars, 2mm. (**d**) Ventricular volume quantification. Bars represent mean ± s.d and circles represent values from individual mice. *p*-values from ANOVA followed by post-test with Tukey’s correction for multiple comparisons.

We hypothesized that activated microglia were key to the development and/or progression of PHH. To clarify the role of microglia in our LPA-induced PHH model, Pexidartinib (PLX3397) was administered to deplete microglia from the developing brain. PLX3397 is a blood-brain barrier-penetrant multi-targeted receptor tyrosine kinase inhibitor of CSF-1R, Kit (c-Kit), and FLT3, capable of eliminating microglia and macrophages [66, 67]. Daily subcutaneous administration of this compound at a 50mg/kg dose can effectively deplete microglia in mice, including mouse pups [68]. To study the effects of microglial depletion on PHH development in this model, PLX3397 (50 mg/kg) was injected daily for 15 days (P1-P15), with LPA injection (5mM) at P7, and tissues were isolated eight days later (P15) (Fig. 5a). The higher dose of LPA was utilized to obtain an unambiguous answer with regard to the role of microglia in the production of ventriculomegaly and neuroinflammation. Immunofluorescence imaging confirmed that PLX3397 administration depleted microglia by P7 (**Online Resource 2**).

Changes to neuroinflammatory cytokines due to the absence of microglia were studied by cytokine array of CSF samples extracted from vehicle-, LPA-, and LPA/PLX3397-injected brains. This analysis revealed that inflammation is sustained, even after microglia depletion, in LPA-injected samples. Nevertheless, PLX3397 administration significantly reduced some cytokine levels to near vehicle baseline levels, including CCL2 (MCP-1), CCL4 (MIP-1β), CCL12 (MCP-5), CCL22 (MDC), IL-16, and IL-17 (Fig. 5b and **Online Resource 3**). PLX3397 induced significant increases in CCL11 (eotaxin) and CX3CL1(fractalkine), chemokines involved in microglial recruitment, and G-CSF and M-CSF, which are both chemokines as well as myeloid growth factors, provided supporting evidence that microglia were involved in the LPA-induced inflammatory response and had been effectively depleted. Two cytokines usually considered pro-inflammatory, CXCL1 (KC) and IFNγ, were also significantly increased. The conventional T helper (Th)-type 2 (Th2) cytokines IL-3, IL-4, IL-5, and IL-10 were all significantly increased, whereas Th1-related IL-16 and Th17 cytokine IL-17 were both significantly decreased. These changes in the cytokine profile indicated that myeloid cell depletion by PLX3397 shifted the neuroimmune environment from a more proinflammatory Th1/Th17 to possibly anti-inflammatory Th2.

The effects of PLX3397-mediated microglial depletion were also assessed on ventricular volume. The lateral ventricles appeared to suffer less damage with PLX3397/LPA as compared to LPA alone. H&E-stained sections and quantification of ventricular volume also revealed a significant reduction of ventriculomegaly (Fig. 5c and d). Thus, depletion of myeloid cells, particularly microglia, demonstrated the key role for these cells in the development of LPA-induced PHH.

## Discussion

We report that activated microglia associated with neuroinflammatory and neurovascular changes are a significant contributor to the development of PHH in an animal model. Pharmacological depletion of microglia by PLX3397 partially prevented enlargement of the lateral ventricles and shifted the neuroinflammatory profile of cells in our LPA-induced hydrocephalus mouse model that phenocopies multiple elements of human PHH and identifies microglia in the promotion of PHH. Proteomic and transcriptomic changes in LPA-induced hydrocephalus may aid biomarker identification in human PHH, consistent with observed changes in CSF concentrations of cytokines and chemokines in the animal model, which overlaps with neuroinflammatory changes observed in human disease. These findings further validate the LPA-induced PHH model and identify potential key drivers of disease that may be therapeutically tractable by medicinal interventions targeting neuroimmune mechanisms.

In contrast to previous studies [30, 31], most of the data presented here were obtained using LPA administered at a lower, more physiologically relevant concentration. This nevertheless resulted in neuroinflammatory responses that were comparable to those induced at higher LPA concentrations in previous models but with a gross phenotype that is more reflective of the human disease state. Interestingly, the proteomic changes that resulted from our LPA exposure model also overlapped with those reported for human disease (Fig. 2E). For example, CCL3 and CCL19 were upregulated at both the proteomic and transcriptomic level in our studies and were also significantly increased in the CSF from human PHH patients [58] (Online Resource 4). The historical names for these chemokines, macrophage inflammatory protein (MIP)-1α and MIP-3β, respectively, provide clues to the potential importance of myeloid cells in the genesis of PHH neuroinflammatory processes.

While proteomic data identified proteins being dysregulated in the CSF, transcriptomic data offered insights into cell types that were affected by LPA. We were able to identify numerous affected cell populations in the mouse brain at P15 and their transcriptomic changes in response to LPA administration. Notably, in response to LPA exposure, astrocytes, vascular cells, and immune cells were the most transcriptionally altered.

Moreover, vascular cells were not only significantly decreased in number, but the number of DEGs observed amongst those remaining were the highest of any cell type. While the reduction in cell population could possibly be attributed to challenges in extracting meningeal layers from hydrocephalic brains, it does not completely explain the increased DEGs in vascular cell types, which may underlie vascular and meningeal dysfunction in PHH. CSF clearance through meningeal lymphatic vessels that drain CSF into the cervical lymph nodes [11] including basal meningeal lymphatics promote CSF clearance [69], and thus our transcriptomic data implicating vasculature dysfunction in PHH paired with meningeal clearance mechanisms are consistent with alteration of this route of CSF clearance in PHH.

The reduction of ventriculomegaly in response to microglial depletion supports their involvement in PHH. While PLX3397-depletion of other myeloid cell types besides microglia [70] could contribute to this effect, the loss of microglia appears most pronounced and reproducible, consistent with the compensatory increase in microglia-recruiting cytokines following PLX3397-induced exposure and cell depletion. Reduced ventriculomegaly, supportive transcriptomic and proteomic data implicate neuroinflammatory microglial changes in the development of PHH, consistent with previous reports [71–73].

Although microglial depletion showed significant PHH-related phenotypic attenuation, it failed to resolve completely the observed changes induced by LPA injection, implicating additional cellular mechanisms. There are several potential explanations for this partial effect that may have clinical relevance for the medical treatment of PHH. While a decrease in many pro-neuroinflammatory markers was observed with microglial removal, there was still significant inflammation as observed in the cytokine panel (Fig. 5b), which may be a result of astrocytic activation in response to LPA injection (Fig. 3e). Astrocytes have key activities in other neurological diseases, such as multiple sclerosis, including those associated with immune processes like those involving the related lysophospholipid sphingosine 1-phosphate (S1P) receptor signaling [74, 75]. Furthermore, while microglial depletion and neuroinflammatory reduction could potentially slow CSF production, damage to ependymal cells and vasculature involved in its clearance could counter effects of microglial removal. Additionally, although often considered to be “anti-inflammatory” in comparison to Th1 or Th17 immune responses, Th2 responses can still generate inflammation. However, Th2 responses are clearly critical for tissue repair in a variety of settings [76]. There is likely a dynamic interplay between CSF production and clearance, matched by a meaningful contribution to each from multiple cell types leading to ventriculomegaly in this PHH model and human PHH, which share phenotypes. It is further notable that LPA and other lysophospholipids like S1P have independent immune effects beyond neuroimmune and neurovascular mechanisms identified here, further raising the potential for novel medical treatments that target neuroimmune mechanisms and LPA signaling pathways in the treatment of PHH and possibly other forms of hydrocephalus.

## Supplementary Material

Supplementary Files

This is a list of supplementary files associated with this preprint. Click to download.

- SanchezPavonOnlineResource13.docx
- SanchezPavonOnlineResource4.xlsx
- SanchezPavonOnlineResource5.xlsx
- SanchezPavonOnlineResource6.xlsx
